# A Simple Technique Based on a Single Optical Trap for the Determination of Bacterial Swimming Pattern

**DOI:** 10.1371/journal.pone.0061630

**Published:** 2013-04-29

**Authors:** Ignacio A. Martínez, Susana Campoy, Meritxell Tort, Montserrat Llagostera, Dmitri Petrov

**Affiliations:** 1 ICFO-Institut de Ciències Fotòniques, Castelldefels, Spain; 2 Departament de Genètica i de Microbiologia, Universitat Autònoma de Barcelona, Bellaterra, Spain; 3 ICREA - Institució Catalana de Recerca i Estudis Avançats, Barcelona, Spain; University of Zurich, Switzerland

## Abstract

Bacterial motility is associated to a wide range of biological processes and it plays a key role in the virulence of many pathogens. Here we describe a method to distinguish the dynamic properties of bacteria by analyzing the statistical functions derived from the trajectories of a bacterium trapped by a single optical beam. The approach is based on the model of the rotation of a solid optically trapped sphere. The technique is easily implemented in a biological laboratory, since with only a small number of optical and electronic components a simple biological microscope can be converted into the required analyzer. To illustrate the functionality of this method, we probed several 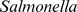 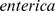 serovar Typhimurium mutants that differed from the wild-type with respect to their swimming patterns. In a further application, the motility dynamics of the 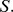 Typhimurium 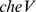 mutant were characterized.

## Introduction

The locomotion of unicellular organisms in aqueous media is mediated by several different mechanisms, among which, the bacterial flagellum is the most thoroughly studied [1]–[4]. This complex structure is composed of several elements that act together to produce helical rotation [5]–[7]. As a semi-rigid helical filament, the flagellum consists of three substructures analogous to an artificial mechanical system: the basal body, which provides an anchor to the bacterial envelope and contains the motor; the filament, which acts as the propeller; and the hook, which connects the basal body to the filament, and acts as a universal joint [2], [7]. Flagella propelled swimming has been thoroughly studied in enteric bacteria such as 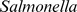 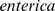 serovar Typhimurium (referred to in the following as 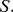 Typhimurium) [4], [7], the bacterium used to develop the method described herein. As other peritrichous bacteria, 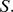 Typhimurium have several flagella over its surface. There are between five and ten flagella several micrometers in length but only about 20 nm in diameter that can rotate both clockwise (CW) and counterclockwise (CCW) [8], [9].

Bacterial motion can be described as a combination of two types of behavior: running and tumbling. During running, the bacterial flagella rotate CCW and form a bundle that pushes the cell along [8], [9]. The bundle collapse when one or more motors of the flagella turn in the CW direction, in which case the bacterium tumbles and reorients itself in another direction [8], [9]. While at the beginning of each run, the cell moves in a random direction, the combination of both movements, i.e., straight lines (running) and random changes (tumbling), allows the cell to explore its environment.

**Figure 1:**
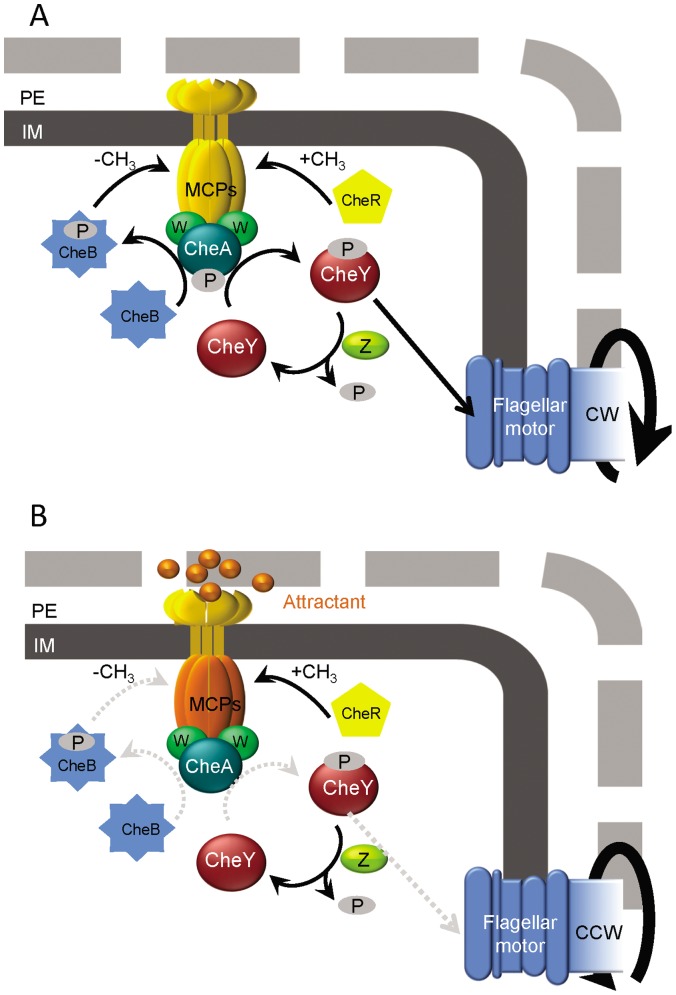
The chemotaxis pathway. (a) When the methyl-accepting chemotaxis proteins (MCPs) are highly methylated or unbound (yellow), CheA is activated by phosphorylation. Once activated, it phosphorylates the CheY and CheB response regulators. CheB-P demethylates the MCPs and the high level of CheY-P interacts with the flagellar motor, increasing the frequency of clockwise (CW) rotation, which causes the cell to tumble. (b) MCPs associated with a ligand (attractant) or less methylated (orange), maintain the CheA in a non-phosphorylated, inactive state. Consequently, the CheB methylesterase is not active and does not demethylate the MCPs. CheR methylation of the MCPs, decreases the sensitivity of these receptors. In addition, the CheY-P levels are reduced, leading to an increase in counterclockwise (CCW) flagellar rotation frequency, causing the cell to run. PE, periplasm; IM, inner membrane; Z, CheZ; W, CheW. The grey discontinuous arrows indicate non-occurring reactions (based on [11], [12]).

Running and tumbling underlie the process of chemotaxis [10] and provide the means by which bacterial cells migrate towards favorable chemicals (attractants) or away from unfavorable ones (repellents). In 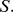 Typhimurium and other bacteria, the presence of chemoattractants/repellents is detected by transmembrane receptors, the methyl-accepting chemotaxis proteins (MCPs) [11], which are associated through the adaptor CheW protein to the CheA kinase [10]. Signal recognition at the chemoreceptor level is determined by degree of MCPs methylation, which in turn reflects the activities of CheR and the methylesterase CheB, and modulates CheA autophosphorylation activity [12]. The phosphorylated kinase (CheA-P) phosphorylates the CheY response regulator, with CheY-P then acting on the flagellar motor. In the absence of CheY-P, the flagella turn CCW and therefore the bacterium runs whereas in the presence of CheY-P the flagella rotate CW and thereby induces tumbling (Fig. 1). When a bacterium senses an attractant gradient, its runs become longer as the number of tumbles decreases, such that the cell migrates up the gradient [13]. Consequently, chemotaxis enables the bacterial cell to find better environments for growth.

Chemotaxis and swimming are also key to the virulence of many pathogens [14]–[16]. In this context, flagellar movement has been intensively studied, mainly via two different strategies: direct visualization of free-moving bacteria and investigations of the rotation of tethered cells. The former typically involves the use of dark-field, differential interface contrast microscopy or, in cells specifically labeled with a fluorescent tag, fluorescence microscopy [9], [17]–[19]. In addition, the motile behavior of cells has been directly studied using microfluidics assays. [20], [21]. Alternatively, in tethered cells assays, the bacteria are fixed to a surface via their flagella [18]. In this case, there is CW or CCW rotation of the basal body depending on the rotational direction of the flagella. Tethered assays normally rely on high-speed video recorders and image processing software programs that quantify swimming speed, the time between the running and tumbling steps, etc.

Among the other experimental approaches used to characterize chemotaxis and flagellar rotation are those based on optical trapping (for example, [22]–[29]). This method exploits the fact that the inner refractive index (cytoplasm) of the bacterium is larger than that of its surrounding medium, such that in the presence of a tightly focused optical beam a radiation force directed towards the beam focus is produced. This results in the immobilization of the bacteria, whose trapped position can be examined using several techniques that provide a microsecond time and nanometer spatial resolutions. The most detailed information on chemotaxis is obtained when flagellar bundle rotation and the counter-rotation of the cell body are analyzed simultaneously. However, until now, this was possible only by using the dual optical trap system proposed in [28] in which a bacterium is trapped at each end by two optical beams polarized orthogonally. The scattered light of both beams is then analyzed separately by two position-sensitive detectors. The Power Spectral Density (PSD) of the output signals from the detectors yields two peaks corresponding to flagellar rotation and body rotation. An analysis of the phase between the detector signals provides a description of the rotation.

An important step in the simplification of this experimental setup (but without a loss of information) was recently reported by [29] where a single optical trap was used to capture a bacterium with a single polar flagellum rotating either CCW or CW. Using a position sensitive photodetector, the authors were able to detect the characteristic frequencies of the bacterium motion by analyzing the PSD of the cell’s position. In addition, they could measure the flagellar motor switching rate under different chemical simulations.

In the following we describe the further refinement of this simple but informative single optical trap technique. We demonstrate that additional information can be obtained if the analysis of the temporal position of the bacterium also includes calculations of the cross-correlation function of the cell trajectory in the plane normal to the optical axis. By applying this approach to a bacterium that uses the run-tumble swimming pattern to navigate within its environment, we were able to study the swimming patterns of different mutant strains across the entire range of bacteria motion within the optical trap. The procedure was validated in dead, running (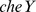, 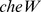), tumbling (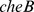 mutant) and wild-type strains of 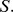 Typhimurium. It was then used, as an example of its potential applications, to identify the role of the CheV protein in the rotation of 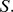 Typhimurium flagella.

## Materials and Methods

### Bacterial Strains, Media, and Growth Conditions

All bacterial strains and plasmids used in this work are listed in Table S1. Except when indicated, the bacteria were grown at 37°C in Luria–Bertani (LB) broth or plates. Ampicillin (100 mg/ml) or chloramphenicol (34 mg/ml) was added to the culture as necessary. Bacteria used in the optical trapping experiments were grown overnight in 2 ml of LB broth supplemented, when needed, with the appropriate antibiotic. Each culture was then diluted 1/10 into LB broth without antibiotic and incubated at 37°C for 1 h. To reduce trap-mediated oxidative damage to the bacterial cells and ensure that a steady level of oxygen was reached during the optical measurements [28], an oxygen scavenging system, consisted of glucose oxidase and catalase at final concentrations of 100 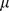
g/mL and 20 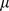
g/ml, respectively, was added at least 2 h before the measurements [30]. The added glucose is a substrate for the oxygen scavenging system and provides the energy needed for swimming in anaerobic conditions [31]. For the optical traping measurements, the culture was further diluted 100-fold in trapping medium (1% Bacto Tryptone, 0.8% NaCl, 2% glucose, 100 mM Tris-Cl, pH 7.5). The use of tryptone broth for the optical trapping experiements is appropriate to obtain reproducible cell motility assays [32]. Dead cells used as controls were prepared by addition of 2% formaldehyde to the culture, with subsequent dilution steps carried out following the same protocol used for the live bacterial cultures.

### Construction of 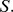 Typhimurium LT2 mutant Derivatives

The 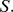 Typhimurium LT2 mutant derivatives used in this work were knockout mutants constructed by the one-step PCR based gene replacement method [33]. All DNA techniques were performed as described elsewhere [34]. The chloramphenicol resistance cassette from the plasmid pKD3 was amplified using suitable 100-nucleotide (nt)- oligonucleotides containing 80-nt stretches homologous to each of the insertion sites (Table S2). The PCR product was DpnI digested and transformated into the 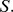 Typhimurium LT2 electrocompetent cells containing the pKOBEGA plasmid [35]. Following selection of the transformant clones, the pKOBEGA plasmid was eliminated by taking advantage of its temperature sensitivity, growing the clones at 42°C. Gene substitution was confirmed by PCR and sequencing. In all cases, the resulting construct was transferred to a wild type 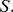 Typhimurium LT2 strain by transduction, using the P22 HT bacteriophage [36]. The absence of the prophage in the selected transductant clones was determined by streaking them onto green plates as previously described [37]. The resulting strains were again verified by PCR and sequencing.

### Optical Setup

**Figure 2:**
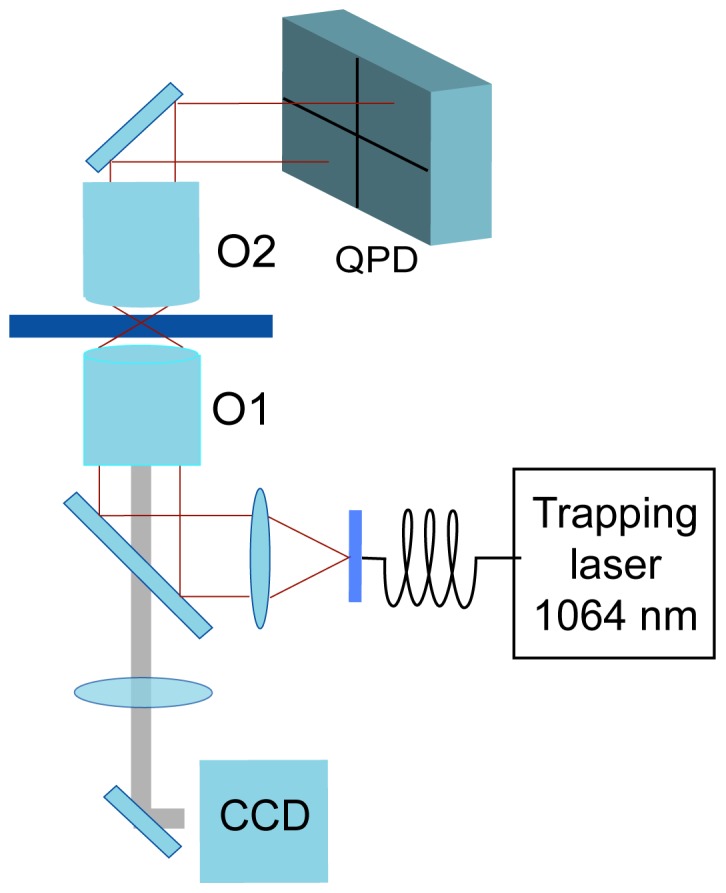
Optical trap layout. A trapping beam (1064 nm) is expanded by a lens in order to overfill the input pupil of the trapping objective, O1, and, hence, increase the trapping force. The position of a trapped object is detected by monitoring the spatial distribution of the trapping beam scattered by the object. This scattered light is collected by an objective, O2, and projected into a quadrant-position detector (QPD).

Optical trapping was carried out using a 1064 nm optical beam from a laser coupled to a single-mode fiber (Avanex) expanded up to 10 mm and then focused by a 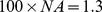 objective (Nikon, CFI PL FL 100X AN 1.30 WD 0.16 mm), as shown in Fig. 2. The optical trapping intensity during the experiments (25 mW) was kept as low as possible in order to immobilize the bacteria while minimizing any oxidative damage. The forward scattered light of the trapping beam was collected by a 40 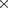 objective, and analyzed using a quadrant-position detector (QPD) (NewFocus 2911). The resulting signals were then transferred to a computer software via an analog to digital conversion card (National Instruments PCI-6120). Cell position data were acquired at a rate of 20 kHz. During the experiments, and especially at the beginning (capture) and end (liberation) of the measurements, videos were recorded using a CCD camera.

**Figure 3:**
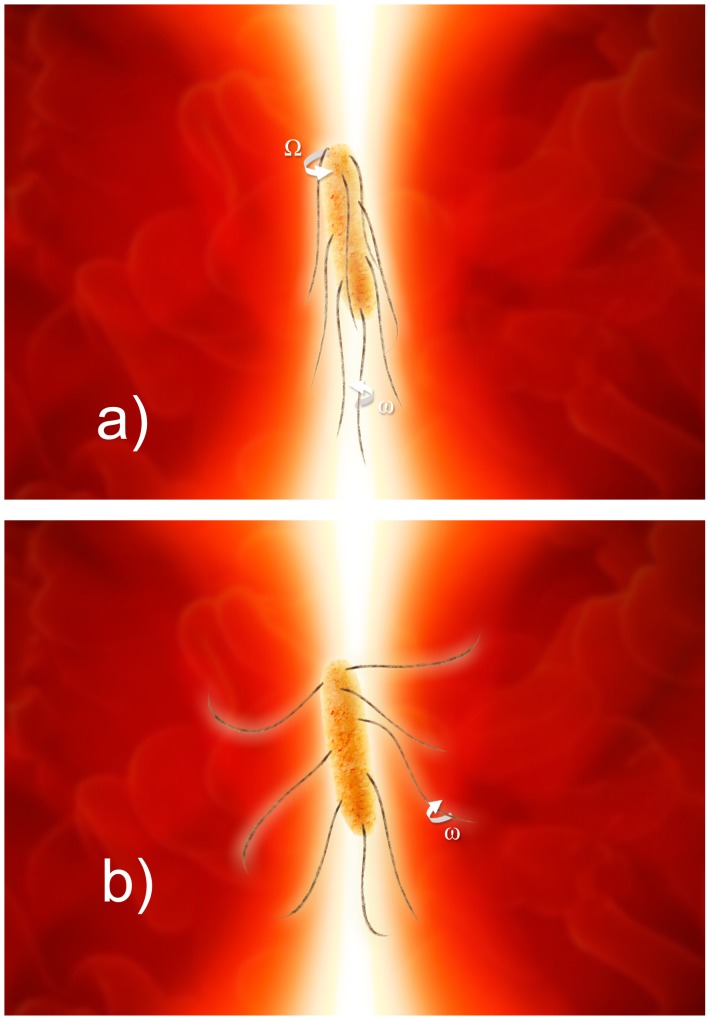
A simplified explanation of the two possible flagellar conformations. On the left is a cell with all flagella rotating CCW and consequently forming a bundle that gives a translation velocity to the bacteria, which runs. The motion of a living bacterial cell is characterized by a body roll with frequency 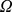, and by flagellar bundle rotation 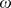. On the right is a tumbling cell with the flagella rotating CW such that no bundle is formed. This process gives extra energy to the cell’s dynamics, but does not produce any rotation around the optical axis of the trapping system.

The optical beam traps those cellular constituents with refractive indices higher than that of the surrounding medium, hence the QPD signals yield information on the momentary position (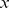 and 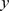 in the plane perpendicular to the trapping beam propagation direction, and 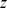 along the propagation direction) of these parts of the bacterial cell. The trajectory of the optically trapped bacteria depends on several factors, both physical (such as the trap stiffness, size of the cell, and viscosity of the medium) and biological (such as the intensity of the flagellar movement). Moreover, cells that are otherwise similar may propel themselves with different velocities, which also affects the trajectories. Due to the cylindrical shape of bacteria, a cell trapped in the single optical beam aligns itself along the optical axis [38]. Besides Brownian motion, the torque produced by the flagella alters the dynamics of the cell due to the possibility of single or double rotations, as shown schematically in Fig. 3.

### Processing of the Experimental Data

The different motility phenotypes are classified based on the study of the trajectory, 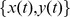, of a single cell optically trapped by the optical tweezers. The starting point of this analysis is the model of the rotation of a solid sphere [39]. Consider a sphere suspended in a liquid medium and confined within a harmonic potential well, where it moves randomly due to thermal excitation. An external torque is exerted on the sphere such that in the absence of any trapping potential the sphere rotates around the 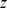-axis with a constant angular velocity due to friction. The angular velocity is determined by the balance between the torque applied to the sphere and the drag torque. The Langevin equations in the overdamped conditions describe the trajectory 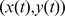 of the sphere as:
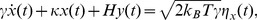
(1)

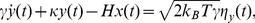
(2)where 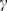 is the friction coefficient, 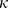 is the trap stiffness, 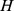 is the torque parameter for an arbitrary rotation, 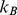 is the Boltzmann constant, 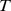 is the temperature, and 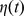 is the white gaussian noise term. The autocorrelation functions (ACF) and the cross-correlation functions (CCF) are calculated from (1) and (2):



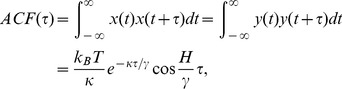
(3)

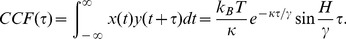
(4)


The 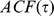 and Power Spectral Density (PSD) form a Fourier pair and both functions can be calculated from the experimental traces of an optically trapped object.

As seen from (3) and (4), in the absence of rotational motions of the trapped object then (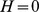), 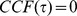, and 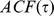 is an exponential function proportional to the viscous force acting on the object.

To apply this model to trapped cells able to rotate in the optical trap, the possible types of rotations must be specified. A flagellated bacterial cell has two main sources of movement: the flagellar rotation and the thermal energy of the bath. Based on the PSD and the traces, two discrete frequencies can appear in the motion of living cells [28], [40]: the body roll characterized by the frequency 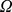 (around 1–10 Hz), and the flagellar bundle rotation with the frequency 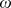 (between 70 and 140 Hz) (see Fig. 3). From the study of the PSD we can infer that the dynamics of a flagellated bacterium are governed more by rotations with frequency 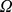 than by those with frequency 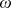, due to a two orders of magnitude difference in the values of the corresponding peaks in the PSD. Therefore, in the equations describing bacterial movement (1) and (2) rotations with the frequency 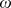, can be neglected, while in the equations (1), (2), (3) and (4).

The CCF is studied for a short correlation time of 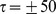
ms, when the entire expression (4) can be approximated by the Taylor series, 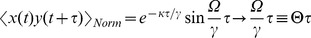, where the physical meaning of 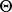 is the angular velocity of the cell around the optical axis.

Accordingly, bacterial cell dynamics can be classified in terms of three different scenarios: dead, running, or tumbling: In a dead cell, the mean value of 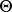 (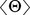) is zero since there is no flagellar movement, but only broadening (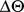) due to thermal fluctuation of the cell. In a running cell, 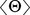 has a non-zero value due to rotation of the cell body as a whole. This value can be positive or negative depending on the orientation of the cell inside the trap. As the number of flagella is small, from 5 to 10, the individual behavior of a single flagellum can affect the statistical distribution of 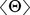, resulting in a 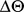 that is larger than in the case of a dead cell. In a tumbling cell, flagella do not form a bundle, thus, while the rotation frequency around the z-axis has a zero average, 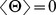, the rotation motion with frequency 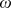 is still present (see 5), causing a larger broadening of the histogram 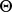 than in occurs with a dead cell.

Data for each bacterial strain were obtained from ten different randomly chosen cells of four distinct biological replicates, and thus a total of 40 cells per strain. The position of each trapped cell was acquired for 1000 s. The entire set of acquired data was then divided into 1-s-blocks (i.e., 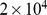 points in each block). For each block of data the CCF was calculated and the dependence of the CCF near 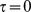 was linearly fit, thereby yielding the value of 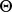. By repeating the protocol for all blocks a histogram of 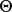 was produced for each measured cell. Then the next cell was trapped and its corresponding histogram was obtained. About 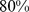 of the histograms were found to behave in a very similar manner. All plots shown below for the wild-type, mutant strains, and dead bacteria, present the 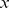 and 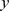 traces, PSD, CCF and 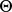 histogram of one trapped cell either from the corresponding bacterial strain or from a dead cell control. In all cases, the selected histograms were within the above-mentioned 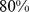.

## Results

In the following, the results obtained using dead bacteria, live wild-type cells, and the three 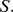 Typhimurium mutant derivatives (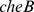, 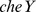, and 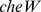, whose behavior has been well-characterized [41]–[43]) are shown. The motion profiles obtained with the proposed technique consist of the ACF and CCF of the trajectories resulting from the different bacterial motility patterns and they provide a reference for further studies.

### Dead Bacteria Pattern

**Figure 4:**
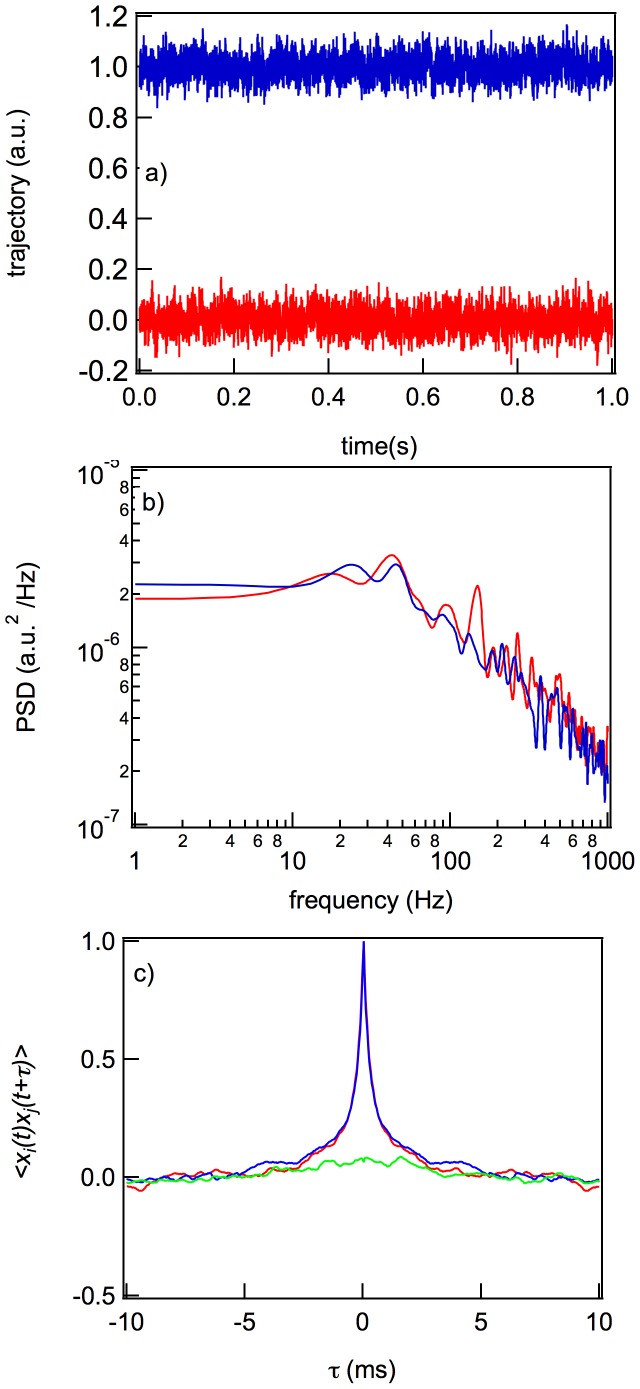
Dynamic characteristics of the motion of a dead bacterium: time-traces of the 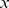 and 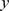 positions during 1 s (a) and their corresponding PSD (b), ACF, and CCF (c). In (a), the 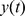 trajectory is shifted in order to improve its visibility. In (c) the red and blue curves are the ACFs of the 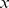 and 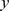 positions, and the green curve is the CCF.

Figure 4 shows the 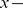 and 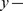 trajectories of the dead bacteria, with their corresponding PSD, ACF, and CCF. For these cells, the PSDs lack the characteristic discrete frequencies seen with live motile bacteria, and they are very similar to the PSDs of the motion of an optically trapped solid sphere thus, the Lorentzian curve is that expected from the experimental conditions. A cross-correlation is also absent from the trajectory since a dead cell does not have any rotational motion. Therefore, as for an optically trapped bead, the ACF depends only on the viscous drag coefficient and the trap stiffness and therefore decays exponentially.

### Tumbling Pattern: The 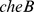 Mutant

The 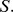 Typhimurium LT2 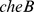 mutant derivative is a knockout mutant of the CheB methylesterase, which in the bacterial chemotaxis pathway controls the level of MCPs methylation (Fig. 1). The 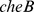 mutants are characterized by a high frequency of tumbling [43].

**Figure 5:**
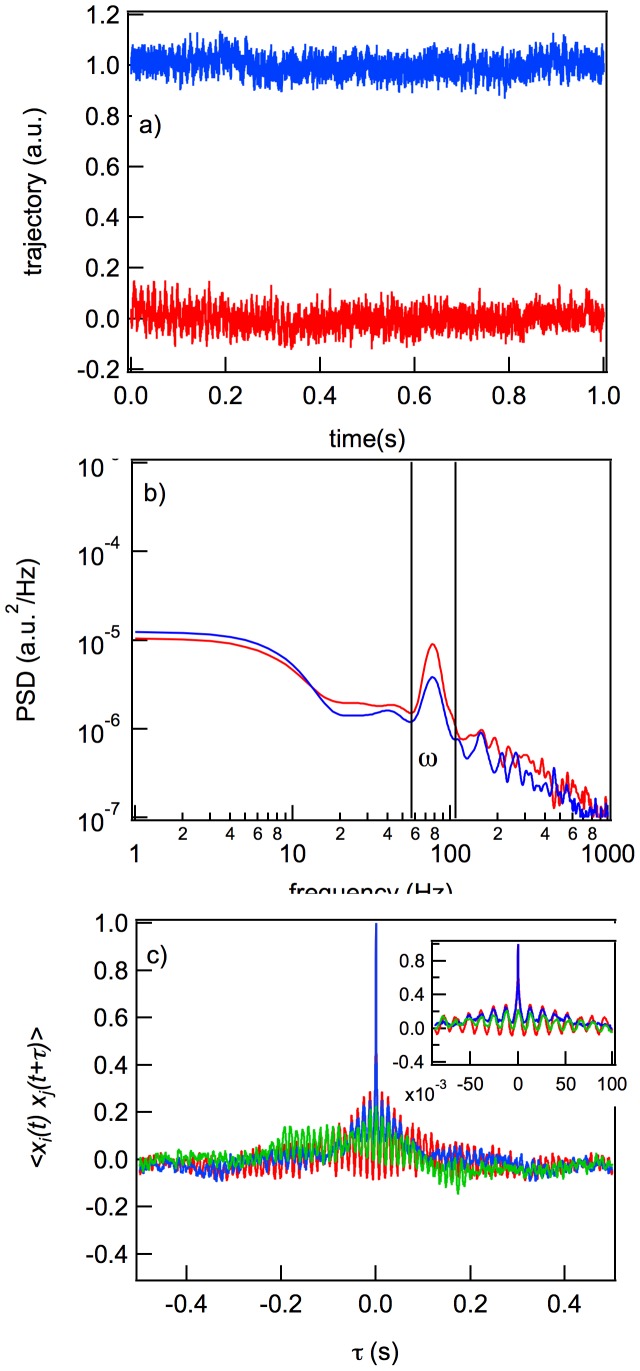
Dynamic characteristics of the 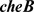 mutant: the trajectories of the 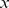 and 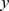 positions during 1 s (a) and their corresponding PSD (b), ACF and CCF (c). The red and blue curves are the trajectories, PSD, and ACF of the 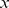 and 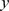 positions, and the green curve is the CCF.

The statistical characteristics of the trajectory of this mutant in the optical trap are presented in Fig. 5. Rotations with angular frequencies in a band near 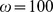 Hz are seen in the PSD, ACF, and CCF, but their presence is also evident even in the trajectories (Fig. 5a). As flagella normally rotate CW, there is no bundle formation. The CCF is not a stable function but instead gives a fluctuating value of 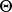. This behavior is related to the continuous chaotic movement of the cell inside the trap.

### Running Pattern: The 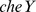 and 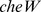 Mutants

**Figure 6:**
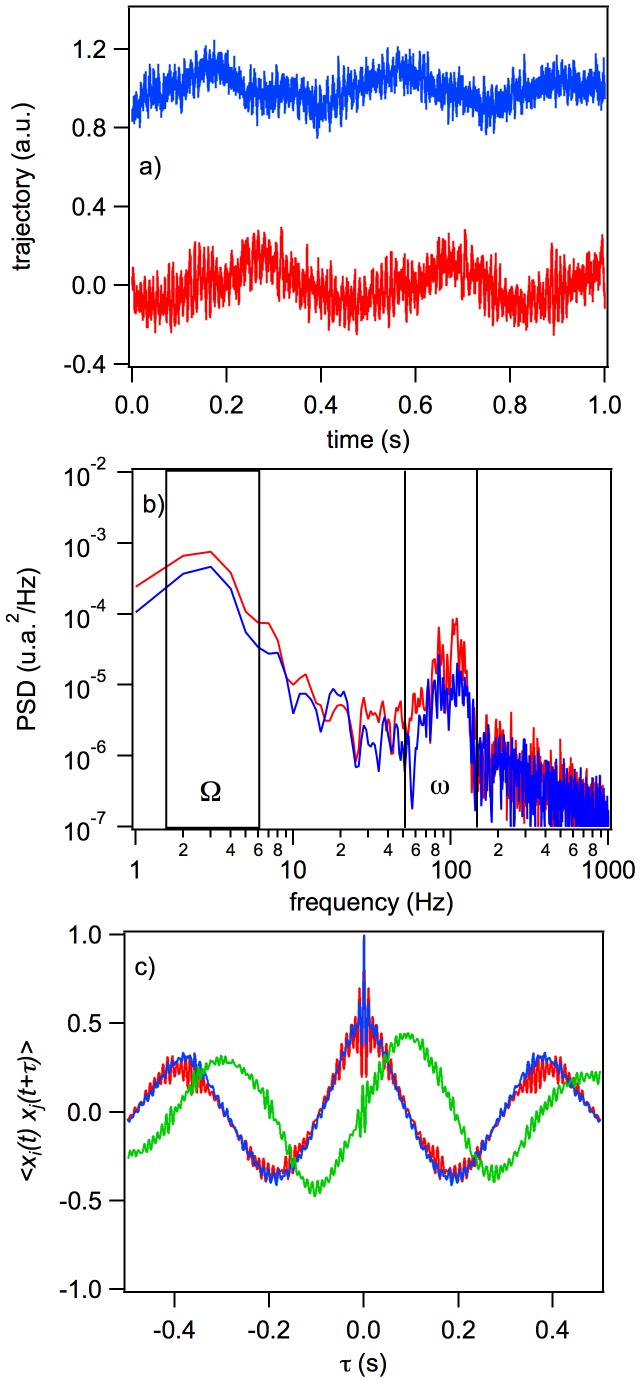
Dynamic characteristics of the 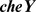 mutant: the trajectories of the 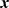 and 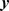 positions during 1 s (a) and their corresponding PSD (b), ACF, and CCF (c). The red and blue curves are the trajectories, PSD, and ACFs of the 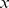 and 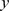 positions, and the green curve is the CCF.

The 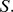 Typhimurium LT2 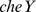 and 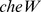 mutants are ideal candidates for exploring running motility, because both present only running pattern of motility not tumbling, what is also known as the smooth swimming profile [42]. In Fig. 6, 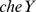 mutant rotations with 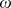 around 100 Hz, and 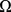 from 5 to 15 Hz are clearly seen, both in the trajectories (Fig. 6a) and in the ACF and CCF (Fig. 6c). The differences in the 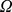 values of the different cells are due to the size differences, which can vary by as much as 30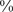 of the length of a typical cell. The same results are observed for the 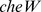 mutants. In both mutants, the dynamics of the bacterial cells inside the trap, with most of the flagella rotating CCW, reflect the rearrangement of all the individual flagellum as a collective bundle positioned at one end of the bacterium. This collective process generates a forward velocity (without the trap it is represented by a quasi-straight line [44]) that is responsible for the global bacterial rotation 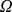. The PSD has two peaks, one centered at 5–15 Hz, corresponding to the cell body roll (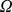) around the trapping point, and the flagella bundle roll around 100 Hz, indicative of rolling by the flagellar bundle (Fig. 6, and data not shown). Accordingly, in these running mutants the histogram of the slope of the CCF for short correlation times clearly points to a different type of dynamic profile from that of tumbling or dead bacteria (See below).

### Wild-type Swimming Pattern

As described above, the swimming pattern of the wild-type strain combines both tumbling and running. In this case, the pattern observed in the representation of the CCF slope for short correlation times is composite of those of the two dynamic types, the tumbling mutant and the running mutant.

**Figure 7:**
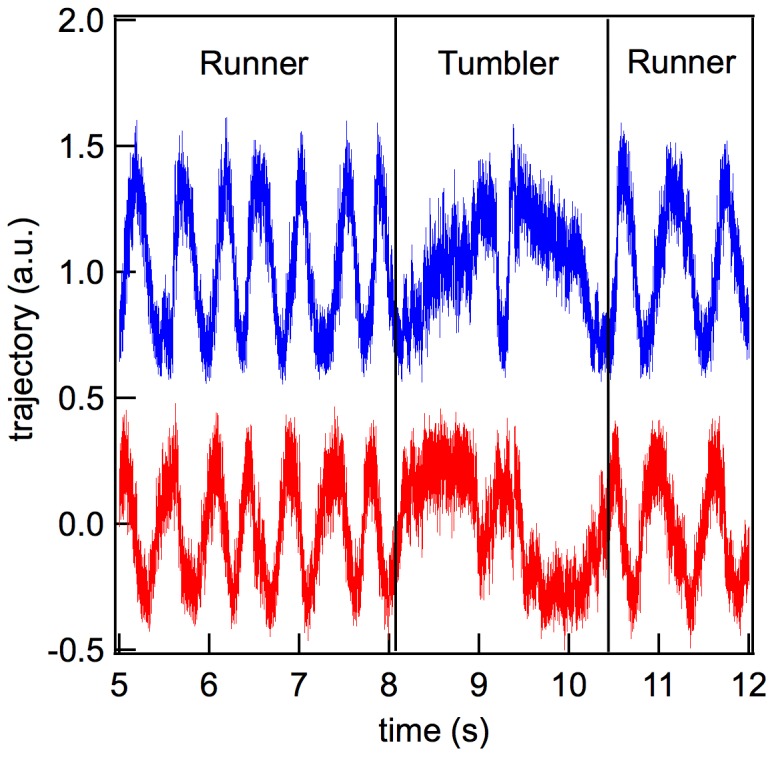
Dynamic characteristics of the 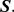 Typhimurium wild-type strain: the 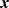 (bottom) and 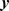 (upper) coordinates of the wild-type bacteria during a 7 s time interval. The the switch of the flagellar motor from the running to the tumbling state is shown. The trace of the 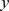 coordinate is shifted in order to avoid overlap with that of the 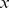 coordinate.

Figure 7 illustrates the traces of the wild-type bacterium, showing the switch from a tumbling to a running state. The traces also allows measurement of the characteristic switching rate of the bacterial motor.

### Histograms of 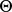


**Figure 8:**
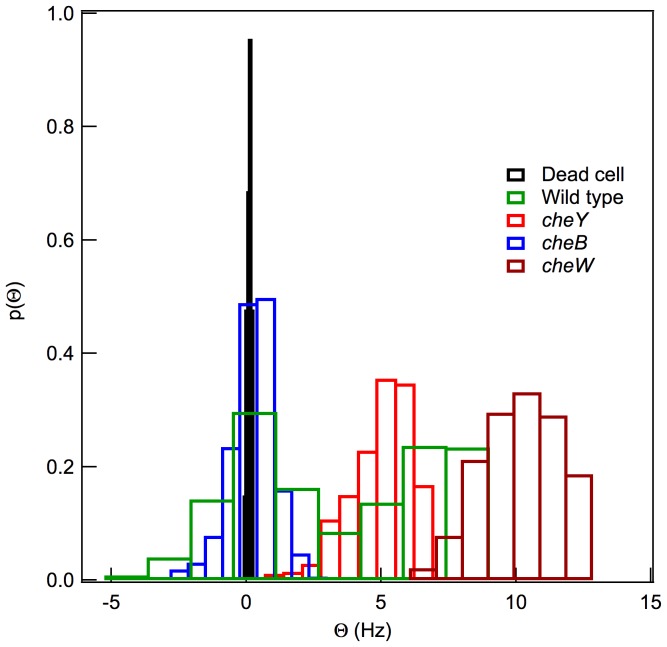
The slope, 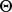, of the CCF near 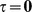 obtained from the trajectories of single optically trapped dead (diamonds), running (circles and crosses, corresponding to 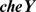 and 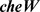 mutants, respectively), 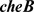 mutant tumbling (squares), and wild-type (triangles) bacteria. The histograms of the distributions can be classified into three main groups: (1) The histograms with a single maximum centered at zero that corresponds to tumbling bacteria (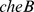 mutant); (2) those with a single maximum not centered at zero correspond to a deterministic rotation associated with the rotation of a solid sphere, as is the case for running bacteria (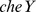 and 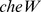 mutants); and (3) a combination of both profiles, as occurs in the wild-type strain.

The results obtained for each bacterial strain with known dynamic properties can now be summarized using the parameter 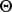, i.e., the slope of the CCF for short correlation times. A comparison of the 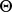 histograms of the four motility patterns (dead cell, tumbling, running, and wild-type cells), distinguishes four types of dynamics (Fig. 8). The widths of the histograms indicate contributions beyond Brownian motion. Accordingly, the histograms from the live cells are different from those of dead bacteria (Fig. 8). In the former, the flagellar frequency does not remain constant, which implies changes in the translational direction [28]. The 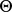 histogram for the wild-type strain is shown in Fig. 8. The correlation functions of this cell should change with time. In dead cells, just the Brownian motion contributes to the width of the histogram, whereas in live cells the contribution of the thermal energy is smaller than that of flagellar motion, resulting in greater broadening of the histograms. Moreover, considering that 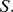 Typhimurium has only five to ten flagella per cell, the histograms also reflect the differential dynamics of a single flagellum since changes therein would affect overall cell movement.

### Identification of the 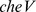 Mutant Swimming Pattern

The function of the CheV protein in the chemotaxis pathway is still not well understood. Although the swimming profile of 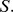 Typhimurium 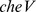 mutants has been characterized [45], the description contradicts earlier published results [41], [43], [46]. For this reason, we chose to apply our newly developed, validated technique to study flagellar rotation in the 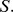 Typhimurium 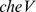 mutant.

**Figure 9:**
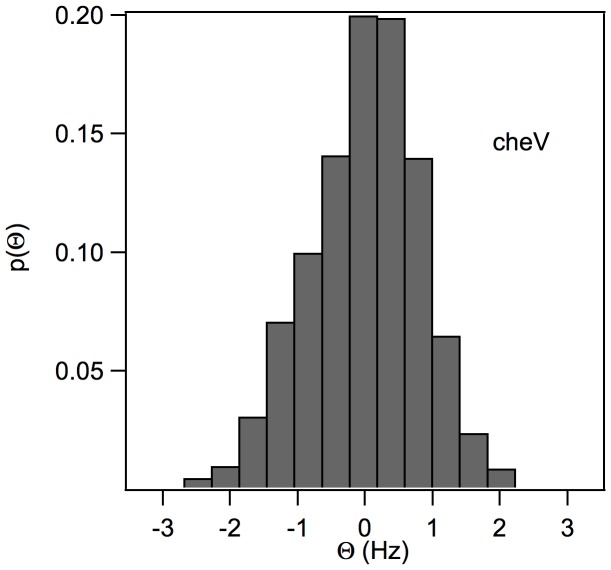
The slope, 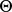, of the CCF near 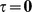, obtained from the trajectories of the optically trapped 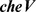 mutants.

A video analysis as well as examination of the ACF and the slope of the CCF for short correlation times (Fig. 9) showed that the 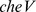 knockout mutant has a tumbling pattern. The absence of the CheV protein modifies the 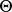 distribution, centering it around zero, with an additional contribution to the 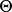 histogram from the random rotations.

## Discussion

The simple single optical trap used in this study to analyze the swimming pattern of 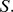 Typhimurium and several mutants can be broadly applied to measure the full range of bacterial motility and alterations thereof. The core of the method is based on our previous study [39], which described the movement of a sub-micron object confined in an optical trap in the presence of a torque exerted on or produced by the object. The single optical trap assay can detect differences in the dynamic properties of cells of the same culture, seen as a change in the mean value of 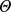, expressed as 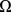, from one cell to another. Our study of ten independent cells from four independent cultures showed that the distribution of the histograms remained very similar, such that the individual phenotype of a single bacterium could be distinguished. The importance of the different parameters is more readily appreciated in the case of the running mutants. For some bacteria, 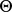 can achieve values close to 100 Hz. It is important to note that in these cases, the widths of the histogram are similar to those obtained for running mutants with only 10 Hz.

Photodamage to the trapped bacterium has been considered [47] given that infrared light in the presence of oxygen produces free radicals inside the cell, which induce death within a short period of time. In our optical trap system, this problem is avoided by including an oxygen scavenging system in the sample preparation medium, which guarantees a constant low level of oxygen and, hence, cell survival during the measurements. The obtained results validate the utility of this optical trap setup to characterize bacterial motility. The analysis of dead cells as well as running, tumbling and wild-type strains of 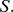 Typhimurium demostrated the possibility to correlate the motility characteristics widely described for these cells with specific 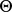 distribution profiles. Thus, the trajectory of the running mutants is characterized by a mean value of 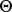 different from zero and that of tumbling mutants by a mean value of 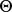 near zero with a certain standard deviation in the latter case due to the random disposition of the flagella and the absence of an equilibrium position.

Changes in bundle formation, from the tail to the head and vice-versa, must also be considered [48]. This effect is unlikely to be stable because the bacteria should work against the radiation pressure in the optical trap, it not being possible to define a stable equilibrium position.

In studying the slope of the CCF for the tumbling bacteria, near 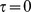, we found that the flagella of these cells rotate in the CW direction and that, like wild-type and running strains, the bacterial dynamics differ from those of dead bacteria (Fig. 4). A dead bacterium behaves as a solid body, therefore, when an extra force is applied, the entire sphere revolves around the trapping point. The sum of these torques will be uniform when averaged over a long time. For a tumbling cell, while the mean value of 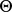 is close to zero, the histogram will be wider than those obtained from a dead bacterium, since the motion detected in the latter case is Brownian motion (Fig. 8).

In addition, for a dead bacterium, there is no flagellar rotation, and therefore neither the ACF nor the CCFs will have a discrete spectrum. The histogram of 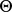 for a dead bacterium has a width much smaller than for a live one, due to the bacterium’s Brownian motion.

Some bacterial species utilize CheV instead of the CheW, whereas in others, including 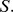 Typhimurium, both proteins are present. In the latter case, the role of CheV is unknown [47]. Most hypotheses regarding CheV function have focused on the two different protein domains: a CheW-like domain and a phosphorylated receiver domain similar to that of CheY. Accordingly, it has been suggested that CheV plays a role in receptor coupling and in the adaptation of the chemotactic response [45], [49], [50]. Furthermore, a 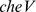 knockout mutant was shown to exhibit the same swimming and chemotactic phenotypes as the wild-type strain [51]. Nevertheless, our single optical trap analysis clearly demonstrated the tumbling phenotype of the 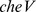 knockout mutant. It should be noted, however, that single optical trap experiments are performed under anaerobic conditions, to avoid cell damage, while in the previously mentioned work the experimental conditions included the presence of oxygen. This is an important consideration because expression of the 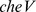 gene is controlled by the global regulators Fnr and ArcA, both of which are involved in O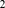 sensing and adaptation [52], [53]. Thus, when the O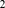 concentration decreases, ArcA and Fnr directly activate 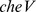 expression, greatly increasing the concentration of CheV inside the cell. Therefore, under anaerobic conditions, such as used in the single optical trap, CheV protein are greatly magnified. In addition, the fact that the 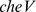 and 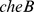 mutants share the tumbling phenotype suggests that the role of CheV in 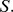 Typhimurium is more closely associated with adaptation more than with receptor coupling. However, further work is needed to elucidate the role of 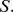 Typhimurium 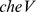 expression in the chemotactic pathway and its relation to the anaerobic metabolism.

### Conclusions

By analyzing the statistical functions derived from following the trajectories 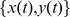 of a bacterium trapped by a single optical beam the different dynamic properties of different bacteria can be distinguished. The approach described herein is based on a model of the rotation of a solid optically trapped sphere. The optical trap technique can be easily implemented in a biological laboratory, since it requires only a small number of optical and electronic parts to convert a simple biological microscope into the required analyzer. In a demonstration of the utility of this method, we determined the motility profile of the 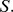 Typhimurium 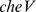 mutant derivative under anaerobic conditions, which case it exhibits tumbling behavior. This observation will contribute to elucidating the role of the CheV protein in the bacterial chemotaxis pathway.

## Supporting Information

Table S1 **Bacterial strains and plasmids used in this work.**
(DOC)

Table S2 **Oligonucleotides used in this work.**
(DOC)
