# Supplementary material for: A Simple Technique Based on a Single Optical Trap for the Determination of Bacterial Swimming Pattern
Source: PLoS One. 2013 Apr 29;8(4):e61630. doi: 10.1371/journal.pone.0061630 (PMC3639288; doi:10.1371/journal.pone.0061630)
Supplement: Table S1 — Bacterial strains and plasmids used in this work. (DOC) [file pone.0061630.s001.doc]

Table S1. Bacterial strains and plasmids used in this work.

| Strain or plasmid | Relevant characteristics | Source or reference |
| --- | --- | --- |
| LT2 | *S.* Typhimurium wild type strain. | Generous gift of Prof. J.L. Ingraham |
| UA1902 | As LT2 but *ΔcheY*. Cm^R^ | This study |
| UA1903 | As LT2 but *ΔcheB*. Cm^R^ | This study |
| UA1905 | As LT2 but *ΔcheW*. Cm^R^ | This study |
| UA1906 | As LT2 but *ΔcheV*. Cm^R^ | This study |
| pKOBEGA | Plasmid Amp^R^, ts | [35] |
| pKD3 | Plasmid Amp^R^, Cm^R^ | [36] |

Table 2. Oligonucleotides used in this work

| Name | Sequence (5’→ 3’) ^a^ | Application |
| --- | --- | --- |
| P1cheB | TGACCCTGGACGTTGAGATGCCGCGTATGGACGGCCTCGATTTTCTGGAAAACTGATGCGCCTGCGACCGATGCCGGTG*gtgtaggctggagctgcttc* | P1 primer used for *S.* Typhimurium *cheB* mutant construction |
| P2cheB | CCTTCACGCTGATTTGACAGAGTTTATTCAGACGCTCGGCAAACGAGCGGGTAAAGCCAGGCGGCATATGCTGCGTAATA*atgggaattagccatggtcc* | P2 primer used for *S.* Typhimurium *cheB* mutants construction |
| cheBeR | TGAAACAGCACATCCACGGA | Lower primer used for *S.* Typhimurium *cheB* mutants confirmation by PCR and sequencing |
| cheBeF | CAGTTGATGATTCCGCGCTA | Upper primer used for *S.* Typhimurium *cheB* mutants confirmation by PCR and sequencing |
| P1cheY | GCTTGGATTTAACAATGTGGAAGAGGCCGAAGACGGCGTCGATGCGCTGAACAAGCTCCAGGCGGGCGGCTTTGGTTTTa*gtgtaggctggagctgcttc* | P1 primer used for *S.* Typhimurium *cheY* mutants construction |
| P2cheY | CATAACCGCTGGCGCCAGCCTGTGCGGCGGCGATAATATTCTCTTTTTTGGCTTCCGCCGTGACCATCAACACGGGTAA*atgggaattagccatggtcc* | P2 primer used for *S.* Typhimurium *cheY* mutants construction |
| cheYeR | CTCTGTTCCGGGATGTTTTCCA | Lower primer used for *S.* Typhimurium *cheY* mutants confirmation by PCR and sequencing |
| cheYeF | TCGTTTGCCGAGCGTCTG | Upper primer used for *S.* Typhimurium *cheY* mutants confirmation by PCR and sequencing |
| P1cheW | GGCGAGCCGTCAGGTCAGGAATTCCTGGTGTTTACACTGGGAAATGAAGAGTACGGCATCGATATCCTGAAAGTGCAGGA*gtgtaggctggagctgcttc* | P1 primer used for *S.* Typhimurium *cheW* mutants construction |
| P2cheW | TATCCAGCAGCGCCATCTCTTCGCTGTTAAGCAGTTTTTCGATATTCACCAGAATCAGCATACGCTCGCCGAGCGCGCCC*atgggaattagccatggtcc* | P2 primer used for *S.* Typhimurium *cheW* mutants construction |
| cheWeR | TCGCTGGCAATGGCGTCATA | Lower primer used for *S.* Typhimurium *cheW* mutants confirmation by PCR and sequencing |
| cheWeF | GTCACGTTGAGATCCAGTCA | Upper primer used for S. Typhimurium cheW mutants confirmation by PCR and sequencing |
| P1cheV | ATCAGGTCATTCCGGTGATTGATTTGCCAGCGGTAGCGGGCTGCAAGCCGGAAACCGGGCTGAATATTTTGCTGATCACC*gtgtaggctggagctgcttc* | P1 primer used for *S.* Typhimurium *cheV* mutants construction |
| P2cheV | CTTCCTGCGCCAGTTGCTGGATTCTCTCCCAGGCATCCTTGCCGGTCACATGCATCTGATGCGGAATTCCCATCGCGTTC*atgggaattagccatggtcc* | P2 primer used for *S.* Typhimurium *cheV* mutants construction |
| cheVeR | CGGCATCTCAAGATCTGTCA | Lower primer used for *S.* Typhimurium *cheV* mutants confirmation by PCR and sequencing |
| cheVeF | GTCTTGGTACATCGCTGCAT | Upper primer used for *S.* Typhimurium *cheV* mutants confirmation by PCR and sequencing |

^a^ P1 and P2 sequences, homologues to the pKD3 plasmid, are represented in lower case italics.
