# Supplementary material for: A Simple Technique Based on a Single Optical Trap for the Determination of Bacterial Swimming Pattern
Source: PLoS One. 2013 Apr 29;8(4):e61630. doi: 10.1371/journal.pone.0061630 (PMC3639288; doi:10.1371/journal.pone.0061630)
Supplement: Table S2 — Oligonucleotides used in this work. (DOC) [file pone.0061630.s002.doc]

Table S2. Oligonucleotides used in this work

| Name | Sequence (5’→ 3’) ^a^ | Application |
| --- | --- | --- |
| P1cheB | TGACCCTGGACGTTGAGATGCCGCGTATGGACGGCCTCGATTTTCTGGAAAACTGATGCGCCTGCGACCGATGCCGGTG*gtgtaggctggagctgcttc* | P1 primer used for *S.* Typhimurium *cheB* mutant construction |
| P2cheB | CCTTCACGCTGATTTGACAGAGTTTATTCAGACGCTCGGCAAACGAGCGGGTAAAGCCAGGCGGCATATGCTGCGTAATA*atgggaattagccatggtcc* | P2 primer used for *S.* Typhimurium *cheB* mutants construction |
| cheBeR | TGAAACAGCACATCCACGGA | Lower primer used for *S.* Typhimurium *cheB* mutants confirmation by PCR and sequencing |
| cheBeF | CAGTTGATGATTCCGCGCTA | Upper primer used for *S.* Typhimurium *cheB* mutants confirmation by PCR and sequencing |
| P1cheY | GCTTGGATTTAACAATGTGGAAGAGGCCGAAGACGGCGTCGATGCGCTGAACAAGCTCCAGGCGGGCGGCTTTGGTTTTa*gtgtaggctggagctgcttc* | P1 primer used for *S.* Typhimurium *cheY* mutants construction |
| P2cheY | CATAACCGCTGGCGCCAGCCTGTGCGGCGGCGATAATATTCTCTTTTTTGGCTTCCGCCGTGACCATCAACACGGGTAA*atgggaattagccatggtcc* | P2 primer used for *S.* Typhimurium *cheY* mutants construction |
| cheYeR | CTCTGTTCCGGGATGTTTTCCA | Lower primer used for *S.* Typhimurium *cheY* mutants confirmation by PCR and sequencing |
| cheYeF | TCGTTTGCCGAGCGTCTG | Upper primer used for *S.* Typhimurium *cheY* mutants confirmation by PCR and sequencing |
| P1cheW | GGCGAGCCGTCAGGTCAGGAATTCCTGGTGTTTACACTGGGAAATGAAGAGTACGGCATCGATATCCTGAAAGTGCAGGA*gtgtaggctggagctgcttc* | P1 primer used for *S.* Typhimurium *cheW* mutants construction |
| P2cheW | TATCCAGCAGCGCCATCTCTTCGCTGTTAAGCAGTTTTTCGATATTCACCAGAATCAGCATACGCTCGCCGAGCGCGCCC*atgggaattagccatggtcc* | P2 primer used for *S.* Typhimurium *cheW* mutants construction |
| cheWeR | TCGCTGGCAATGGCGTCATA | Lower primer used for *S.* Typhimurium *cheW* mutants confirmation by PCR and sequencing |
| cheWeF | GTCACGTTGAGATCCAGTCA | Upper primer used for S. Typhimurium cheW mutants confirmation by PCR and sequencing |
| P1cheV | ATCAGGTCATTCCGGTGATTGATTTGCCAGCGGTAGCGGGCTGCAAGCCGGAAACCGGGCTGAATATTTTGCTGATCACC*gtgtaggctggagctgcttc* | P1 primer used for *S.* Typhimurium *cheV* mutants construction |
| P2cheV | CTTCCTGCGCCAGTTGCTGGATTCTCTCCCAGGCATCCTTGCCGGTCACATGCATCTGATGCGGAATTCCCATCGCGTTC*atgggaattagccatggtcc* | P2 primer used for *S.* Typhimurium *cheV* mutants construction |
| cheVeR | CGGCATCTCAAGATCTGTCA | Lower primer used for *S.* Typhimurium *cheV* mutants confirmation by PCR and sequencing |
| cheVeF | GTCTTGGTACATCGCTGCAT | Upper primer used for *S.* Typhimurium *cheV* mutants confirmation by PCR and sequencing |

^a^ P1 and P2 sequences, homologues to the pKD3 plasmid, are represented in lower case italics.
